# Acyclovir resistance in herpes simplex virus type I encephalitis: a case report

**DOI:** 10.1007/s13365-016-0489-5

**Published:** 2016-10-27

**Authors:** M. Bergmann, R. Beer, M. Kofler, R. Helbok, B. Pfausler, E. Schmutzhard

**Affiliations:** Department of Neurology, Neurocritical Care Unit, Medical University Innsbruck, Anichstrasse 35, 6020 Innsbruck, Austria

**Keywords:** Acyclovir resistance, Herpes simplex virus type I, Encephalitis, Foscarnet

## Abstract

Acyclovir resistance is rarely seen in herpes simplex virus (HSV) type I encephalitis. Prevalence rates vary between 0.5 % in immunocompetent patients (Christophers et al. 1998; Fife et al. 1994) and 3.5–10 % in immunocompromised patients (Stranska et al. 2005). We report a 45-year-old, immunocompetent (negative HIV antigen/antibody testing), female patient, without previous illness who developed—after a febrile prodromal stage—aphasia and psychomotor slowing. Cerebral magnetic resonance imaging (cMRI) showed right temporal and insular T2-hyperintense lesions with spreading to the contralateral temporal lobe. Cerebrospinal fluid (CSF) analysis yielded lymphocytic pleocytosis and elevated protein level. Polymerase chain reaction testing for HSV type I showed a positive result in repeat lumbar puncture. HSV type I encephalitis was diagnosed and intravenous acyclovir treatment was initiated (750 mg t.i.d.). Acyclovir treatment was intensified to 1000 mg t.i.d., due to clinical deterioration, ongoing pleocytosis and progression on cMRI 5 days after initiation of antiviral therapy. In parallel, acyclovir resistance testing showed mutation of thymidine kinase gene at position A156V prompting foscarnet therapy (60 mg t.i.d.). Patient’s condition improved dramatically over 2 weeks. Acyclovir resistance is rare but should be considered in case of clinical worsening of patient’s condition. To our knowledge, this is the first report of acyclovir resistance in HSV type I encephalitis of an immunocompetent and previously healthy patient in Austria.

## Introduction

Clinically suspected acyclovir resistance of herpes simplex virus (HSV) type I was first described in 1982 (Burns et al. 1982; Crumpacker et al. 1982; Sibrack et al. 1982) with reported prevalence rates of 0.5 % in immunocompetent patients (Christophers et al. 1998; Fife et al. 1994) and 3.5–10 % in immunocompromised patients.(Stranska et al. 2005) Even higher prevalence rates (25 %) of acyclovir resistance have been published in bone marrow or allogenic hematopoietic stem cell transplant recipients (Danve-Szatanek et al. 2004; Morfin et al. 2004). Of note, acyclovir resistance in HSV type I encephalitis is rare (Gateley et al. 1990; Schulte et al. 2010).

## Report of a case

**Fig. 1:**
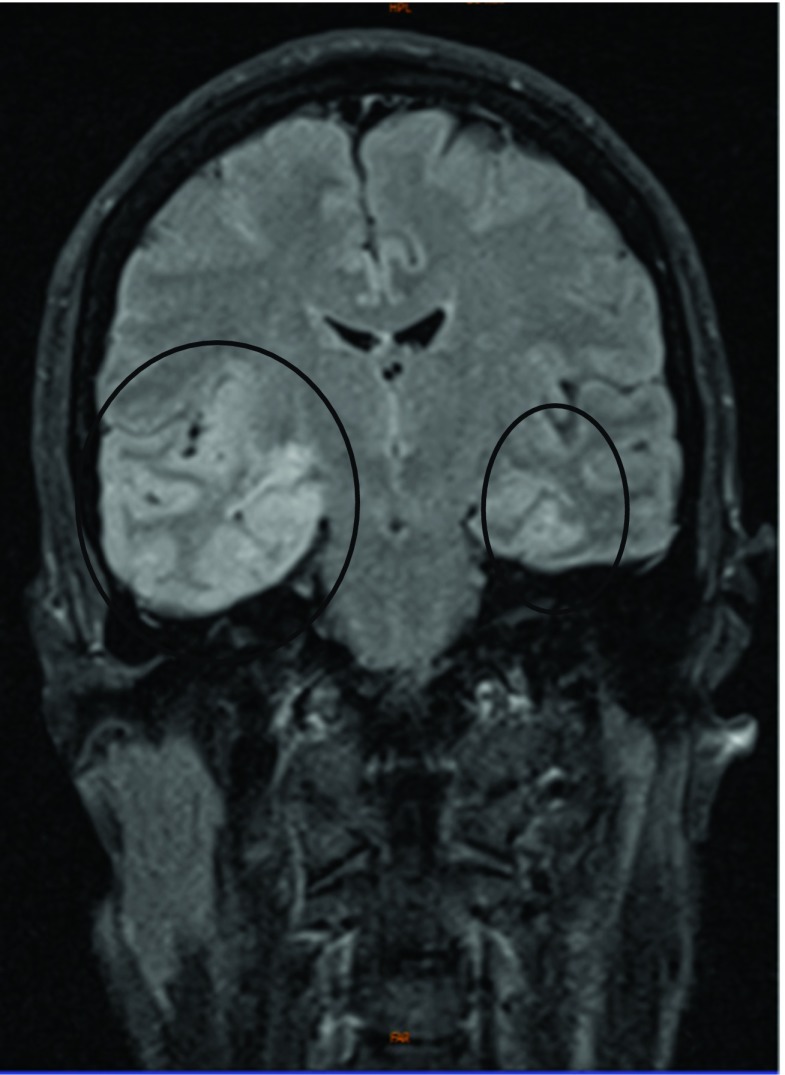
ᅟTurbo inversion recovery magnitude (TIRM) cMRI scan taken at day three after beginning of symptoms

**Fig. 2:**
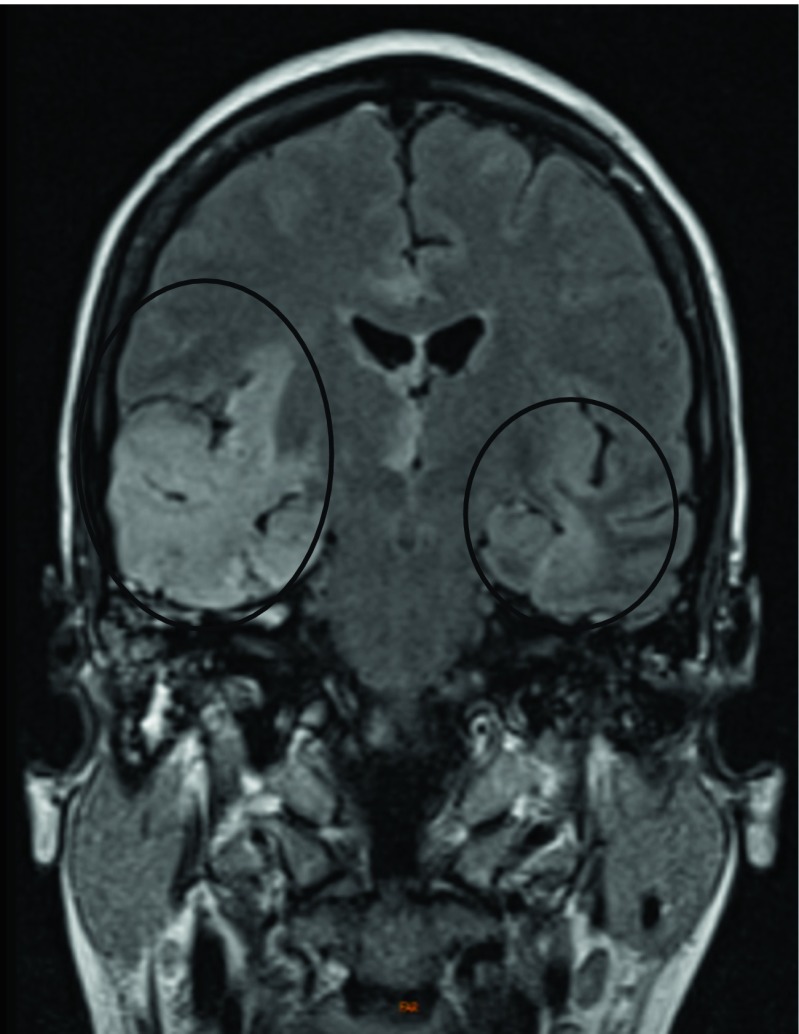
ᅟFollow-up cMRI scan performed 5 days later from first cMRI

A 45-year-old woman suffering from right frontal headache, fever, diarrhea, and vomiting, without previous illness, was admitted to a district hospital. She was evaluated for systemic viral disease with negative testing for putative pathogens including human immunodeficiency virus (HIV). White blood cell classification revealed left shift with a rise of neutrophils (neutrophils 8.3 billion cells/L (G/L), total leucocytes 10 G/L, lymphocytes 1.3 G/L, monocytes 0.38 G/L, eosinophils 0 G/L, basophils 0 G/L). Blood immunoglobulin levels were normal. Three days later, she developed psychomotor slowing and aphasia and, finally, became stuporous, but had no obvious neck stiffness. Cerebrospinal fluid (CSF) examination revealed lymphocytic pleocytosis (271 cells/μL; reference <4 cells/μL), mild elevated protein (82 mg/dL; normal range <50 mg/dL), but normal glucose concentration. Cerebral magnetic resonance imaging (cMRI) showed T2-hyperintense lesions, mainly involving the right temporal lobe and the insula, as well as the frontal and contralateral temporal regions (Fig. 1). On the following day, a negative PCR testing for HSV type I in the previously drawn CSF sample was reported. However, based upon the patient’s clinical presentation, CSF, and cMRI findings, HSV type I encephalitis was still suspected and intravenous acyclovir (750 mg t.i.d.) had been instituted immediately after the diagnostic procedures. Despite prompt initiation of antiviral therapy, the patient developed repetitive generalized tonic-clonic seizures and respiratory insufficiency requiring mechanical ventilation. Consequently, the patient was transferred to our neurological intensive care unit (ICU). Repeat cMRI 5 days after initiation of acyclovir treatment showed progressing T2-hyperintense lesions (Fig. 2). Further, repeat CSF examination yielded ongoing lymphocytic pleocytosis (243 cells/μL) and this time positive PCR result for HSV type I. To overcome potential inadequate penetration of acyclovir into the intrathecal compartment, the dose of acyclovir was increased to 1000 mg t.i.d. In addition, to rule out suspected resistance to acyclovir, genotype sequencing of thymidine kinase and DNA polymerase genes was performed. Molecular biology testing confirmed mutation of thymidine kinase gene at position A156V. Immediately after positive resistance testing, foscarnet was added at a dose of 60 mg t.i.d. The combination of increased acyclovir dose and the initiation of foscarnet led to a marked improvement of the patient’s condition. The third lumbar puncture performed at day 24 after hospitalization revealed a reduction of CSF pleocytosis to 18 cells/μL and HSV type I DNA was no longer detectable in the CSF.

The patient was then weaned off the ventilator and extubated. At transferal to a specialized neurological rehabilitation facility 1 month after hospital admission the patient still showed residual aphasia, left-sided hemiparesis, and cognitive dysfunction. She left rehabilitation center with modified Rankin scale of 3 (moderate disability).

## Discussion

The patient suffered from HSV type I encephalitis and was initially treated with an intravenous acyclovir dose of 750 mg t.i.d. Time from onset of symptoms to treatment was approximately 72 h. Clinical and neuroimaging deterioration together with the development of intracranial hypertension prompted further investigations, particularly for potential presence of acyclovir resistance—even in the case of an immunocompetent patient, however, no further investigation with respect to primary immunodeficiency (Joshi et al. 2009) was carried out. We were able to confirm a mutation (A156V) of the viral enzyme thymidine kinase which converts the prodrug acyclovir to acyclovir monophosphate. A156V mutation is a well-known cause of HSV type I drug resistance.

Acyclovir is the first-line treatment for HSV infections and requires activation via phosphorylation to the triphosphate form. Initially, phosphorylation takes place through thymidine kinase encoded by the human herpesvirus 1 UL23 gene. Further phosphorylation steps occur through cellular thymidylate kinases. Activated acyclovir triphosphate is a competitive inhibitor of the viral DNA polymerase and results in chain termination. Long-term prophylaxis with acyclovir may lead to drug resistance, especially in immunocompromised patients (Andrei and Snoeck 2013). The HSV may become resistant to acyclovir via mutation of the viral thymidine-kinase gene, mediating approximately 95 % of cases of acyclovir resistance, or via mutation of the DNA polymerase gene. Resistance to acyclovir can be established by clinical observation, phenotyping (testing the susceptibility of a viral isolate to different drug doses), or genotyping by the identification of specific mutations linked with drug resistance (Andrei and Snoeck 2013).

Foscarnet is a direct inhibitor of the virus DNA polymerases and an alternative drug for the treatment of HSV resistant patients (Piret and Boivin 2011; Safrin et al. 1991; Schulte et al. 2010). Alternative therapeutic approaches have been introduced recently, including viral polymerase inhibition by brincidofovir (CMX001), valomaciclovir, and n-methanocarbathymidine. Another option is the inhibition of the helicase primase complex with pritelivir (AIC316) or amenamevir (ASP2151) (Scott and Prichard 2014). Finally, we do not yet know whether increasing the dose of acyclovir or the initiation of foscarnet led to an improvement of our patient’s medical condition.

## Conclusion

Acyclovir resistance in HSV type I encephalitis is rare in immunocompetent patients (Gateley et al. 1990; Schulte et al. 2010) but should be considered in cases of clinical deterioration despite antiviral therapy and persistent virus load in the CSF. Here, we report the first case of an immunocompetent patient with acyclovir resistant HSV type I encephalitis in Austria. Genotype sequencing reliably reveals mutations of thymidine kinase as a cause of acyclovir resistance. In our case, increasing the dose of acyclovir during the assessment of resistance and the timely initiation of foscarnet proved life-saving; however, the delay of the appropriate therapy certainly has contributed to medium-term morbidity.
